# Transcriptome Analysis Reveals That Ascorbic Acid Treatment Enhances the Cold Tolerance of Tea Plants through Cell Wall Remodeling

**DOI:** 10.3390/ijms241210059

**Published:** 2023-06-13

**Authors:** Qianyuan Fu, Hongli Cao, Lu Wang, Lei Lei, Taimei Di, Yufan Ye, Changqing Ding, Nana Li, Xinyuan Hao, Jianming Zeng, Yajun Yang, Xinchao Wang, Meng Ye, Jianyan Huang

**Affiliations:** 1 National Center for Tea Plant Improvement, Key Laboratory of Biology, Genetics and Breeding of Special Economic Animals and Plants, Ministry of Agriculture and Rural Affairs, Tea Research Institute, Chinese Academy of Agricultural Sciences, Hangzhou 310008, China; fuqianyuan@tricaas.com (Q.F.); wanglu317@tricaas.com (L.W.); leilei@tricaas.com (L.L.); ditaimei@tricaas.com (T.D.); yeyufan528@163.com (Y.Y.); chqding@tricaas.com (C.D.); nanali@tricaas.com (N.L.); haoxy@tricaas.com (X.H.); zengjm@tricaas.com (J.Z.); yjyang@tricaas.com (Y.Y.); xcw75@tricaas.com (X.W.); 2 Key Laboratory of Tea Science in Universities of Fujian Province, College of Horticulture, Fujian Agriculture and Forestry University, Fuzhou 350002, China; lili9885@126.com; 3 College of Food Science, Southwest University, Chongqing 400715, China

**Keywords:** ascorbic acid, cold stress, ROS, cell wall, tea plants

## Abstract

Cold stress is a major environmental factor that adversely affects the growth and productivity of tea plants. Upon cold stress, tea plants accumulate multiple metabolites, including ascorbic acid. However, the role of ascorbic acid in the cold stress response of tea plants is not well understood. Here, we report that exogenous ascorbic acid treatment improves the cold tolerance of tea plants. We show that ascorbic acid treatment reduces lipid peroxidation and increases the Fv/Fm of tea plants under cold stress. Transcriptome analysis indicates that ascorbic acid treatment down-regulates the expression of ascorbic acid biosynthesis genes and ROS-scavenging-related genes, while modulating the expression of cell wall remodeling-related genes. Our findings suggest that ascorbic acid treatment negatively regulates the ROS-scavenging system to maintain ROS homeostasis in the cold stress response of tea plants and that ascorbic acid’s protective role in minimizing the harmful effects of cold stress on tea plants may occur through cell wall remodeling. Ascorbic acid can be used as a potential agent to increase the cold tolerance of tea plants with no pesticide residual concerns in tea.

## 1. Introduction

Being sessile, plants need to cope with different environmental stresses such as cold, heat, drought, and salt to ensure growth and survival. Due to climate change, extreme weather events are likely to become more frequent and intense. Cold stress is one of the major environmental factors that limits plant growth, development, crop productivity, and geographical distribution [1]. During evolution, plants have acquired a set of sophisticated mechanisms that enable them to respond to environmental challenges on multiple levels. For instance, plants respond to cold stress at the cellular level by changing their cell wall construction, membrane permeability, cell cycle, and cell division [2,3]. At the physiological and molecular level, various substances or protective proteins are changed during cold stress, such as the accumulation of carbohydrates, amino acids, ascorbic acid (AsA), and flavonoids. These substances are involved in the regulation of osmotic potential, ice crystal formation, the stability of cell membranes, and reactive oxygen species (ROS) scavenging in cold-stressed plants [4,5,6].

AsA, also known as vitamin C, is an important water-soluble antioxidant in plants and plays a crucial role in plant growth, development and stress responses [7,8,9]. AsA content is regulated by AsA biosynthesis and recycling pathways. Four AsA biosynthetic pathways have been proposed, including D-mannose/L-galactose, L-gulose, D-galacturonate, and *myo*-inositol [10,11,12,13]. The D-mannose/L-galactose pathway is considered the dominant AsA biosynthesis pathway in plants. The ascorbate–glutathione cycle is considered the main AsA regeneration pathway. In this pathway, ascorbate peroxidase (APX) uses AsA as an electron donor for the reduction of hydrogen peroxide (H_2_O_2_), monodehydroascorbate is reduced to AsA by monodehydroascorbate reductase (MDHAR), and dehydroascorbate is reduced to AsA by dehydroascorbate reductase (DHAR), respectively. By reducing oxidized glutathione (GSSG) to glutathione (GSH), glutathione reductase (GR) enables the removal of ROS and the regeneration of AsA in plants [7].

Previous studies showed that mutations or the overexpression of genes in the AsA biosynthesis and regeneration pathways affected AsA content and stress tolerance. For instance, the GDP-mannose pyrophosphorylase (GMP, VTC1) *vtc1-1* mutant displays one-third of the amount of AsA compared to wild-type plants and is hypersensitive to atmospheric ozone [14,15]. The overexpression of a tomato *GMPase* in tobacco increases the content of AsA, thereby leading to an increase in tolerance to temperature stress [16]. However, the gene silencing of *OsVTC1-1* promotes a defense response in rice and enhances resistance to the rice blast fungus [17]. The overexpression of the tomato GDP-mannose-3,5-epimerase genes *SlGME1* and *SlGME2* increases AsA content and enhances tolerance to salt and cold stress [18]. *VTC2* and *VTC5*, which are two homologous genes, encode GDP-L-galactose phosphorylase in *Arabidopsis* [19,20,21]. The *vtc2vtc5* double mutant has almost no GDP–L-galactose phosphorylase activity and displays significantly decreased AsA levels as well as growth arrest [19]. In addition, the overexpression of *PbDHAR2* from *Pyrus sinkiangensis* in tomato increases the AsA level and enhances tolerance to salt and chilling stresses [22]. A mechanistic study reveals that *PbrMYB5* acts upstream of *PbDHAR2*, and the overexpression of *PbrMYB5* in *Nicotiana benthamiana* also increases the AsA level and enhances tolerance to cold stress [23].

Plant cell walls provide many important functions, including regulating plant morphogenesis and architecture, providing mechanical support for the plant body, transporting water and nutrients, and protecting the plant from biotic and abiotic challenges [24]. The plant cell walls are composed of polysaccharide polymers such as cellulose, hemicellulose, pectin, lignin, and glycoproteins [25]. Cellulose is synthesized by the cellulose synthase complex (CSC), which is made of different cellulose synthase proteins [26]. Genetic studies reveal that the *Arabidopsis* CSC contains three CESA proteins (CESA1, CESA3, and either CESA2, CESA5, CESA6, or CESA9) [27]. A mutation in *AtCesA8*, one of the 10 *Arabidopsis CesAs*, results in increased tolerance to osmotic and drought stress [28]. However, the mutation of a cellulose synthase-like protein, AtCSLD5, makes *Arabidopsis* vulnerable to salt stress and osmotic stress [29]. Hemicelluloses are polysaccharides in plant cell walls that include xyloglucans, xylans, mannans, glucomannans, and β-(1,3; 1,4)-glucans [30]. Xylans are the most abundant hemicellulosic polymers in vascular plants [24]. Pectins are identified as critical components in plant responses to either heat or cold temperature stress in a variety of species, including *Arabidopsis* (*Arabidopsis thaliana*), rice (*Oryza sativa*), and soybean (*Glycine max*) [31].

Tea plants (*Camellia sinensis* (L.) Kuntze) are an important economic woody crop, and the new shoots that sprout in spring are the raw material for manufacturing various types of tea products, such as green tea, black tea, white tea, yellow tea, dark tea, and oolong tea. Tea is a popular beverage that has several health benefits for humans, such as anti-inflammatory, anti-cancer, and anti-obesity effects, which depend on the biochemical properties and bioavailability of the components in tea [32]. As a perennial evergreen plant, tea plants experience low temperatures (cold), which happen in winter and early spring throughout their lifetime. Low temperatures in the winter affect the survival of tea plants, whereas low temperatures in the spring have a negative impact on tea production and quality. Using natural metabolites to improve tea plants’ cold tolerance is an effective way of avoiding pesticide residue and ensuring tea safety. In addition to catechin, the AsA level was also increased during cold acclimation in tea plants in our previous comparative metabolomic analysis [6]. However, whether AsA can increase cold stress tolerance or not in tea plants is unclear. Therefore, in this study, we investigated the effects of AsA on the cold tolerance of tea plants. In addition, to explore the possible mechanisms through which AsA increases cold tolerance, transcriptome analysis was conducted on the young shoots of tea plants. Our study will aid in understanding the mechanism underlying the AsA-mediated cold stress response and provide evidence that AsA is a potential natural metabolite for increasing the cold tolerance of tea plants with no pesticide residual concerns in tea.

## 2. Results

### 2.1. AsA Treatment Improves the Cold Stress Tolerance of Tea Plants

**Figure 1:**
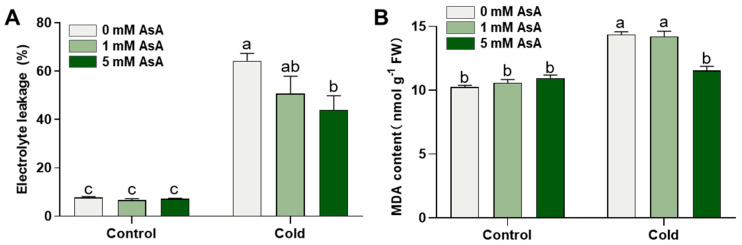
AsA treatment improves the cold stress tolerance of tea plants. (**A**) The relative electrolyte leakage assay of young shoots with one bud and two leaves before and after cold stress. Data are mean ± SEM of five biological replicates. Control: normal temperature treatment; Cold: cold stress treatment. Different letters indicate significant differences as assessed by two-way ANOVA followed by Tukey’s HSD comparisons (*p* < 0.05). (**B**) The MDA content of young shoots before and after cold stress. Data are mean ± SEM of three biological replicates. Control: normal temperature treatment; Cold: cold stress treatment. Different letters indicate significant differences as assessed by two-way ANOVA followed by Tukey’s HSD comparisons (*p* < 0.05).

To investigate the role of AsA in tea plants’ cold stress response, we pretreated tea plants with AsA at concentrations of 1 mM and 5 mM. AsA was applied for two days, once per day. Twenty-four hours after the last application of AsA, the plants were either subjected to cold stress (−3 °C for 2.5 h) or maintained under normal temperature conditions (25 °C). Then, the relative electrolyte leakage was measured in the young shoots with one bud and two leaves. Cold stress significantly increased the electrolyte leakage in the young shoots (Figure 1A), suggesting that cold stress impairs plant cell membrane integrity. In addition, the 5 mM AsA treatment caused a significant decrease of 31.62% in electrolyte leakage, while the 1 mM AsA treatment had a slight effect on electrolyte leakage. In contrast, AsA treatment had no effect on electrolyte leakage without cold stress (Figure 1A).

To examine whether AsA treatment protects plants from cold-induced lipid peroxidation, we measured the content of malondialdehyde (MDA). In comparison to normal temperature control, cold stress elevated MDA levels by 40.10% (Figure 1B). A 5 mM AsA treatment significantly reduced the MDA level, with a 19.64% lower level than the 0 mM AsA treatment control (Figure 1B), indicating that AsA can improve the cold stress tolerance of tea plants.

### 2.2. AsA Treatment Protects Photosynthesis of Tea Plants

**Figure 2:**
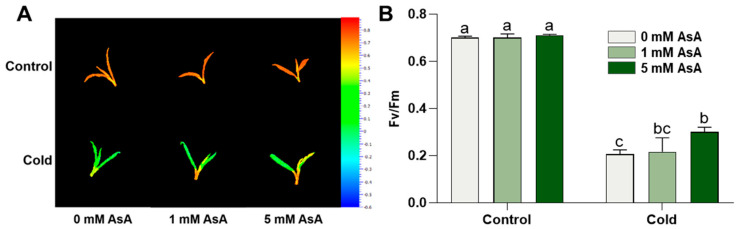
AsA treatment protects the photosystem of tea plants. (**A**) Pseudo-color image of the young shoots before and after cold stress treatment. Pseudo-color gradient shown on the right side of the image ranges from −0.6 (blue) to 0.8 (red). Control: normal temperature treatment; Cold: cold stress treatment. (**B**) Fv/Fm value in young shoots before and after cold stress treatment. Data are mean ± SEM of five biological replicates. Control: normal temperature treatment; Cold: cold stress treatment. Different letters indicate significant differences as assessed by two-way ANOVA followed by Tukey’s HSD comparisons (*p* < 0.05).

To determine whether AsA has a protective role in photosynthesis, we measured Fv/Fm, which reflects the maximum quantum efficiency of photosystem II (PSII) photochemistry. AsA application did not affect Fv/Fm under normal conditions (Figure 2A,B). When compared to normal conditions, cold stress decreased Fv/Fm from 70% to 20.6% (Figure 2B). After cold stress, the 5 mM AsA treatment caused a significant increase in Fv/Fm, which was 45.63% higher than the 0 mM AsA treatment control (Figure 2A,B), suggesting that AsA treatment could protect photosystem II of the tea plants under cold stress.

### 2.3. Transcriptome Assembly and Differentially Expressed Genes Analysis

To explore how AsA treatment enhances the tea plant’s cold stress tolerance, we performed mRNA sequencing (RNA-seq) analysis. Twenty-four hours after the application of AsA, the tea branches were subjected to cold stress treatment (C), whereas the normal temperature controls were not (N). Twelve RNA-seq libraries were constructed, and these were divided into four groups: CO (tea plants treated with MiliQ-H_2_O and subjected to cold treatment), CV (tea plants treated with 5 mM AsA and subjected to cold treatment), NO (tea plants treated with MiliQ-H_2_O and kept at normal temperature), and NV (tea plants treated with 5 mM AsA and kept at normal temperature). More than 85% (86.44–88.6%) of the clean reads in each sample were uniquely mapped to the tea plant ‘Longjing 43’ reference genome (Table S2).

**Figure 3:**
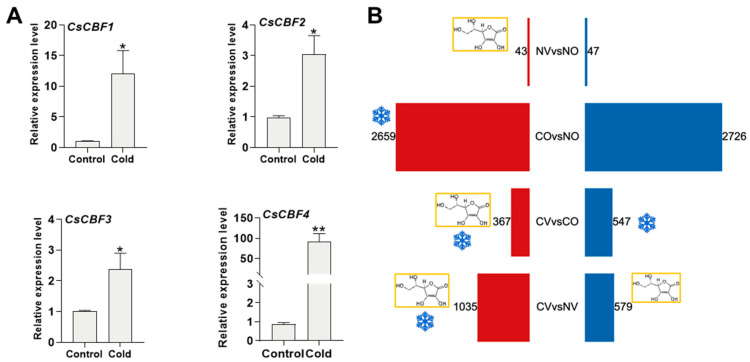
The expression pattern of *CsCBF* genes and the number of DEGs under different comparisons. (**A**) The qPCR analysis of *CsCBF1, CsCBF2, CsCBF3*, and *CsCBF4* in the tea young shoots with one bud and two leaves. *CsPTB* gene was used as the internal control. Control: normal temperature treatment; Cold: cold stress treatment. The *x*-axis indicates the different conditions. The *y*-axis is the relative expression level, and the expression level of each gene under control condition was set to one. Values are mean ± SEM of four biological replicates. Asterisks indicate significant differences (* *p* < 0.05, ** *p* < 0.01), as determined by Student’s *t*-test. (**B**) The number of DEGs is identified in the four comparisons (CO vs. NO, CV vs. CO, CV vs. NV, and NV vs. NO). The red color indicates up-regulated genes; the blue color indicates down-regulated genes. C: cold stress treatment; N: normal temperature treatment; V: treated with 5 mM AsA; O: treated with MiliQ-H_2_O; NV: treated with 5 mM AsA and kept at normal temperature; NO: treated with MiliQ-H_2_O and kept at normal temperature; CO: treated with MiliQ-H_2_O and cold stress; CV: treated with 5 mM AsA and cold stress.

We confirmed that the cold stress treatment was successful by examining the induction of cold stress marker genes. Consistent with previous studies [6,33], we found that *CsCBF1*, *CsCBF2*, *CsCBF3*, and *CsCBF4* had low expression levels under normal temperature conditions and were induced by cold stress treatment (Figure 3A). In the non-cold stress treatment, 90 genes, including 43 up-regulated and 47 down-regulated genes, were differentially expressed genes (DEGs) in the AsA treatment (NV) compared with the normal temperature control (NO) (NV vs. NO) (Figure 3B, Table S3). After cold stress treatment, there were 5385 DEGs, including 2659 up-regulated and 2726 down-regulated genes in the comparison between CO and NO (Figure 3B, Table S3). In the CV vs. CO comparison, there were 914 DEGs, with 367 up-regulated and 547 down-regulated genes (Figure 3B, Table S3). Comparing CV to NV revealed 1614 DEGs, including 1035 up-regulated and 579 down-regulated genes (Figure 3B, Table S3).

### 2.4. Gene Ontology (GO) Enrichment Analysis of DEGs

**Figure 4:**
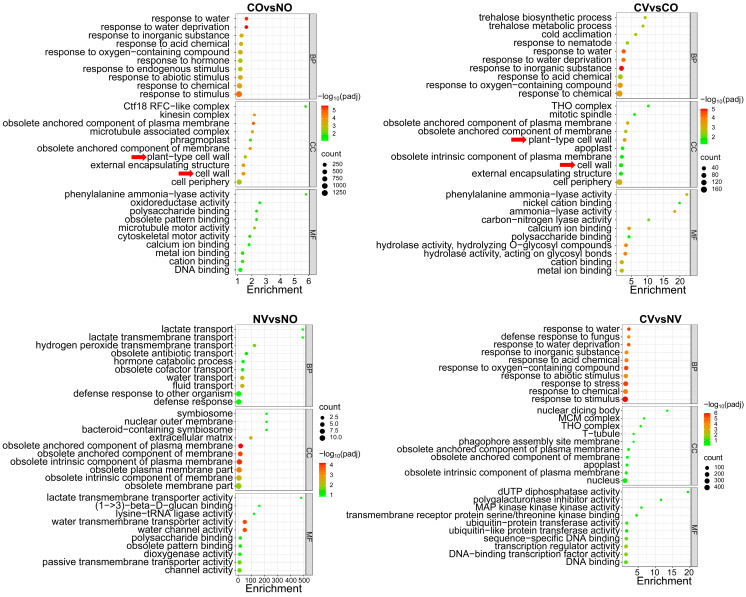
Gene ontology enrichment analysis of DEGs in the CO vs. NO, CV vs. CO, NV vs. NO, and CV vs. NV comparisons. The *p*-value of the enrichment analysis is represented by color; red indicates a small *p*-value, and green indicates a large *p*-value. The number of genes is indicated by the size of the bubble. The red arrows indicate the GO terms related to the cell wall. NV: treated with 5 mM AsA and kept at normal temperature; NO: treated with MiliQ-H_2_O and kept at normal temperature; CO: treated with MiliQ-H_2_O and cold stress; CV: treated with 5 mM AsA and cold stress.

To determine the possible mechanism underlying the AsA-enhanced tea plants’ cold stress response, we performed GO enrichment analyses on the 76 DEGs (novel genes are not included) from the NV vs. NO comparison, 5052 DEGs from the CO vs. NO comparison, 849 DEGs from the CV vs. CO comparison, and 1525 DEGs from the CV vs. NV comparison. The top GO term enrichments among all DEGs in each pair-wise comparison are listed in Figure 4. In the NV vs. NO comparison, many of the GO terms related to transport, such as lactate transport, H_2_O_2_ transmembrane transport, and water transport, were enriched. The top GO term enrichments of DEGs from the CO vs. NO comparison (reflecting the response of cold stress alone) are related to biological processes, such as the response to water, the response to endogenous stimulus, and the response to abiotic stimuli, with cellular components related to the cell wall. For the CV vs. CO comparison (reflecting the effect of AsA treatment on cold stress response), the top GO terms are for biological processes similar to the CO vs. NO comparison, and with the cellular components related to plant-type cell wall and cell wall as well. The GO term analysis of the different comparisons suggests that AsA-enhanced cold tolerance may be achieved via the modulation of distinct transport and plant cell wall components.

### 2.5. AsA Treatment Negatively Regulates the Expression of Genes Involved in ROS Scavenging

**Figure 5:**
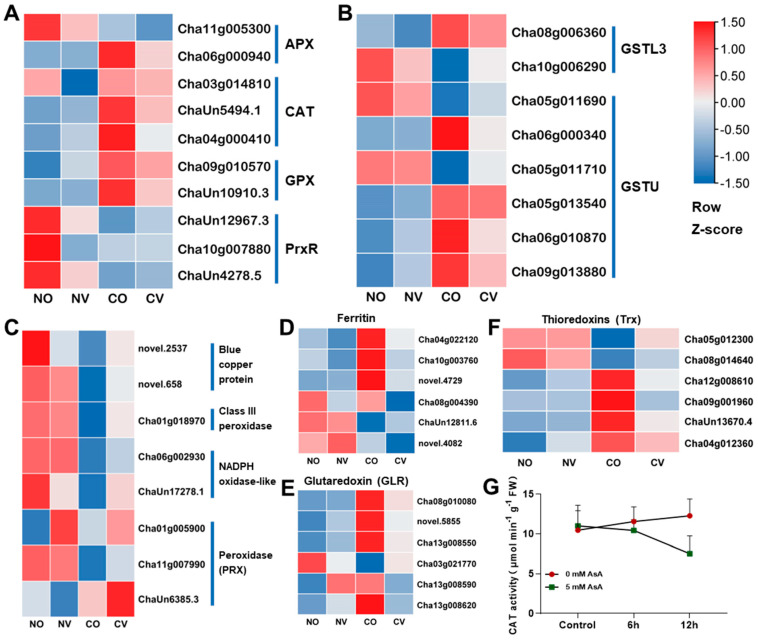
AsA treatment negatively regulates the expression of the genes involved in ROS scavenging. (**A**) The heatmap shows the expression pattern of the *APX*, *CAT*, *GPX*, and *PrxR* genes in different conditions. (**B**) The heatmap shows the expression pattern of the *GSTL3* and *GSTU* genes. (**C**) The heatmap shows the expression pattern of the genes encoding blue copper protein, class III peroxidase, NADPH oxidase-like, and peroxidase (PRX). (**D**–**F**) The expression profile of other DEGs related to ROS scavenging. NO: treated with MiliQ-H_2_O and kept at normal temperature; NV: treated with 5 mM AsA and kept at normal temperature; CO: treated with MiliQ-H_2_O and cold stress; CV: treated with 5 mM AsA and cold stress. (**G**) CAT activity assay of young shoots before and after cold stress treatment. The *x*-axis indicates the different conditions. The *y*-axis is CAT activity. Data are mean ± SEM of five biological replicates.

To investigate how AsA treatment affects the ROS-scavenging system, we analyzed the expression of genes encoding superoxide dismutase (SOD), ascorbate peroxidase (APX), catalase (CAT), glutathione peroxidase (GPX), and peroxiredoxin (PrxR). Without cold stress, the exogenous application of AsA did not change the expression levels of the genes involved in ROS scavenging (Figure 5, Table S4). Under cold stress, *CsAPX3* (*Cha06g000940*), *CsCAT2* (*Cha04g000410* and *ChaUn5494.1*), *CsGPX2* (*Cha09g010570*), and *CsGPX6* (*ChaUn10910.3*) were differentially expressed in the CO vs. NO comparison (Figure 5A, Table S4). Interestingly, most of the up-regulated genes had lower expression levels in the CV compared to the CO condition (Figure 5A–F, Table S4), suggesting that the exogenous application of AsA negatively affects the expression of genes involved in ROS scavenging. Then, we measured CAT enzyme activity. In accordance with gene expression, CAT enzyme activity increased under cold stress but decreased with AsA application; however, it did not reach a statistically significant level (Figure 5G).

### 2.6. AsA Treatment Negatively Regulates AsA Biosynthesis Gene Expression under Cold Stress

**Figure 6:**
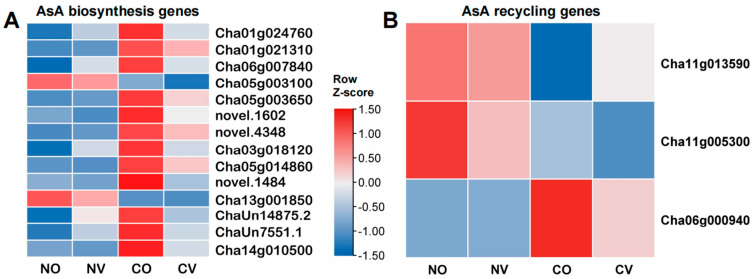
AsA negatively regulates biosynthesis genes expression under cold stress. (**A**) The heatmap shows the expression pattern of the DEGs involved in the biosynthesis of AsA. (**B**) The heatmap shows the expression pattern of the DEGs involved in AsA recycling. NO: treated with MiliQ-H_2_O and kept at normal temperature; NV: treated with 5 mM AsA and kept at normal temperature; CO: treated with MiliQ-H_2_O and cold stress; CV: treated with 5 mM AsA and cold stress.

Our previous comparative metabolic analysis revealed that cold stress increased AsA content in tea plants [6]. In order to determine which genes are responsible for the increase in AsA level, we analyzed the expression levels of the genes involved in the AsA biosynthesis and recycling pathways. Without cold stress, the exogenous application of AsA did not change the expression levels of the genes involved in the AsA biosynthesis and recycling pathways (Table S5). Under cold stress, multiple genes that are involved in AsA biosynthesis, such as *CsPGI1* (*Cha01g024760*), *CsPMI* (*Cha01g021310*), *CsPMM* (*Cha06g007840*), *CsVTC2* (*Cha03g018120*, *Cha05g014860*, and *novel.1484*), *CsVTC5* (*novel.1602* and *novel.4348*), *CsMIOX1* (*ChaUn14875.2* and *ChaUn7551.1*), and *CsMIOX2* (*Cha14g010500*), were up-regulated in the CO vs. NO comparison (Figure 6, Table S5). Interestingly, all of these genes had lower expression levels with AsA treatment (CV vs. CO) (Figure 6, Table S5), suggesting that AsA treatment negatively affects the biosynthesis of AsA under cold stress. The genes involved in AsA recycling, such as *CsAO* (*Cha11g013590*) and *CsAPX1* (*Cha11g005300*), were down-regulated by cold stress (CO vs. NO) (Figure 6, Table S5). However, in the CV vs. CO comparison, *CsGME* (*Cha05g003100*) and *CsGalLDH* (*Cha13g001850*), which are involved in AsA biosynthesis, were down-regulated, while *CsAPX3* (*Cha06g000940*), which is involved in AsA recycling, was up-regulated by cold stress (Figure 6, Table S5).

### 2.7. AsA Treatment Affects the Expression of Cell Wall Remodeling-Related Genes

**Figure 7:**
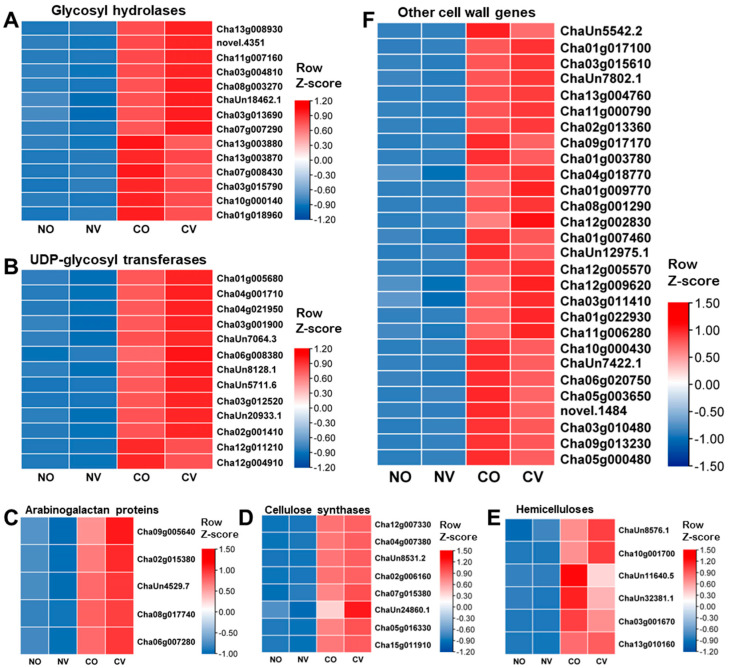
Expression analysis of the genes related to cell wall remodeling. (**A**) The heatmap shows the expression pattern of 14 genes encoding glycosyl hydrolases. (**B**) The heatmap shows the expression pattern of 13 genes encoding UDP-glycosyl transferases. (**C**) The heatmap shows the expression pattern of 5 genes encoding arabinogalactan proteins. (**D**) The heatmap shows the expression pattern of 8 genes encoding cellulose synthases. (**E**) The heatmap shows the expression pattern of 6 genes related to hemicelluloses. (**F**) The heatmap shows the expression pattern of other genes involved in the modification and degradation of the cell wall. NV: treated with 5 mM AsA and kept at normal temperature; NO: treated with MiliQ-H_2_O and kept at normal temperature; CO: treated with MiliQ-H_2_O and cold stress; CV: treated with 5 mM AsA and cold stress.

Under abiotic stress, the expression of the genes involved in the modification and degradation of cell walls is altered, resulting in a remodeling of the cell wall [34]. Among the 367 up-regulated genes in the CV vs. CO comparison, 50 of these genes are associated with cell wall modification and degradation, with the majority of these genes belonging to the arabinogalactan protein, cellulose synthase, glycosyl hydrolase, and UDP-glucosyl transferase gene families (Figure 7, Table S6). In detail, the fifty up-regulated genes consisted of five arabinogalactan protein coding genes (*Cha09g005640*, *Cha02g015380*, *ChaUn4529.7*, *Cha08g017740*, and *Cha06g007280*), eight cellulose synthase genes (*Cha12g007330*, *Cha04g007380*, *ChaUn8531.2*, *Cha02g006160*, *Cha07g015380*, *ChaUn24860.1*, *Cha05g016330*, and *Cha15g011910*), two β-1,3-glucanase genes (*ChaUn8576.1* and *Cha10g001700*), eight glycosyl hydrolase genes (*Cha13g008930*, *novel.4351*, *Cha11g007160*, *Cha03g004810*, *Cha08g003270*, *ChaUn18462.1*, *Cha03g013690*, and *Cha07g007290*), eleven UDP-glucosyl transferase genes (*Cha01g005680*, *Cha04g001710*, *Cha04g021950*, *Cha03g001900*, *ChaUn7064.3*, *Cha06g008380*, *ChaUn8128.1*, *ChaUn5711.6*, *Cha03g012520*, *ChaUn20933.1* and *Cha02g001410*), four β-galactosidase genes (*Cha01g017100*, *Cha03g015610*, *ChaUn7802.1* and *Cha13g004760*), two β-xylosidase genes (*Cha11g000790* and *Cha02g013360*), three pectin methylesterase genes (*Cha01g009770*, *Cha08g001290*, and *Cha12g002830*), two plasmodesmata callose-binding protein coding genes (*Cha12g009620* and *Cha03g011410*), two expansin genes (*Cha01g022930* and *Cha11g006280*), one pectin lyase gene (*Cha04g018770*), one pectinacetylesterase gene (*Cha12g005570*), and one glucan synthase gene (*Cha13g010160*). The twenty-four down-regulated genes are also related to cell wall modification and degradation, including one beta-glucosidase gene (*ChaUn5542.2*), one beta-xylosidase gene (*Cha09g017170*), one UDP-XYL synthase gene (*Cha01g003780*), three xyloglucan endotransglycosylase genes (*ChaUn11640.5*, *ChaUn32381.1* and *Cha03g001670*), six glycosyl hydrolase genes (*Cha13g003880*, *Cha13g003870*, *Cha07g008430*, *Cha03g015790*, *Cha10g000140*, and *Cha01g018960*), one pectin methylesterase gene (*Cha01g007460*), one UDP-D-glucuronate 4-epimerase gene (*ChaUn12975.1*), two UDP-Glycosyltransferase genes (*Cha12g011210* and *Cha12g004910*), three galactose oxidase genes (*Cha10g000430*, *ChaUn7422.1*, and *Cha06g020750*), two GDP-L-galactose phosphorylase genes (*Cha05g003650* and *novel.1484*), one galactose mutarotase gene (*Cha03g010480*), and two beta-amylase genes (*Cha09g013230* and *Cha05g000480*) (Figure 7, Table S6). Thus, AsA may increase cell wall thickness or maintain cell wall integrity under cold stress, increasing the cold tolerance of tea plants.

## 3. Discussion

Cold stress is one of the harshest abiotic stresses that negatively affects both the yield and quality of tea [35]. Therefore, it is crucial to develop approaches for improving the cold tolerance of tea plants [6,33,36,37,38,39,40]. Natural metabolites have recently been widely used to improve plant growth, crop yield, and stress resistance [41,42,43]. In this study, we found that cold stress caused lipid peroxidation and decreased the Fv/Fm of tea plants. We also showed that AsA had a protective role against cold stress in tea plants by regulating AsA biosynthesis, ROS scavenging, and cell wall remodeling.

ROS are oxygen-containing molecules that are more chemically reactive than O_2_, and they are a natural byproduct of plant cellular metabolism. However, when plants are exposed to a variety of stresses, large amounts of ROS accumulate, disrupting the homeostasis of ROS. High concentrations of ROS can cause oxidative injury to cells by damaging proteins, lipids, and other macromolecules. Thus, it is necessary to maintain ROS levels within the optimal range for plant health [44]. In order to cope with the oxidative stress induced by unfavorable environmental conditions, plants have evolved effective ROS-scavenging systems that can be enzymatic or non-enzymatic, which work synergistically and interactively to neutralize ROS [45]. The enzymatic ROS-scavenging systems consist of superoxide dismutases (SODs), peroxidases (PODs), catalases (CATs), ascorbate peroxidase (APX), and glutathione peroxidase (GPX). Non-enzymatic antioxidants include ascorbate acid, GSH, flavonoids, tocopherol, and alkaloids [45]. Consistently, cold stress induced the expression levels of *CsAPX3*, *CsCAT2*, *CsGPX2*, and *CsGPX6*, as well as CAT enzyme activity (Figure 5G). Although comparable studies on various plant species have been published with similar conclusions, the potential molecular mechanisms are different. For instance, previous studies demonstrated that the exogenous application of AsA enhanced cold stress tolerance by activating various antioxidant enzyme activities such as CAT, GPX, APX and SOD [46]. Other investigations, however, have reached different explanations. Under salt stress, the application of AsA reduces the enzyme activity in leaves of Canola (*Brassica napus* L.), but has no effect on the enzyme activity in roots [47]. Exogenous ascorbic acid induces chilling tolerance in tomato, and AsA substantially reduces CAT and heat shock protein gene expression [41]. Therefore, it is essential to investigate the function and molecular mechanism of AsA in the cold stress response of tea plants. To the best of our knowledge, this is the first study demonstrating that the exogenous application of AsA enhances the cold stress tolerance of tea plants. In this study, we discovered that ascorbic acid treatment down-regulates the expression of genes involved in ascorbic acid biosynthesis and ROS scavenging. One of the explanations is that, in order to maintain a non-toxic and steady-state level of ROS, plants that experience the pretreatment of AsA reduce the expression levels of other genes that participate in ROS scavenging since AsA directly eliminates several types of ROS.

The remodeling of cell wall biosynthesis is a common response to environmental changes [48]. When plants are subjected to biotic or abiotic challenges, their cell wall composition and structure, as well as individual components, are modified. The appropriate and timely remodeling of the cell wall is critical to the plant’s survival strategy in adverse situations. Plant cell walls represent a dynamic network that is not easy to analyze, and as a consequence, the majority of studies about cell wall modifications under abiotic stress focus primarily on the genes putatively involved in cell wall metabolism, while the changes in the cell wall itself are only barely studied [34]. In our RNA-seq analysis, we found that many of the genes related to cell wall remodeling, such as cellulose synthases, arabinogalactan proteins, glycosyl hydrolases, and the UDP-glucosyl transferase genes, were differentially expressed under cold stress (Figure 7, Table S6). Cold stress up-regulates cellulose synthase genes, which are found in many plant species, including rice [49] and cotton [50]. Arabinogalactan proteins (AGPs) are a superfamily of highly glycosylated hydroxyproline-rich glycoproteins that are commonly found in most plant species. Studies of AGPs reveal that AGPs are involved in the stress response. For instance, many periplasmic arabinogalactan proteins are up-regulated by salt stress [51]. GhAGP31 is a non-classical arabinogalactan protein from cotton, and its expression is induced by cold stress [52]. The overexpression of *GhAGP31* in yeast and *Arabidopsis* significantly improves the freezing tolerance of yeast cells and the cold tolerance of *Arabidopsis* seedlings [52]. As a result of the large number of DEGs associated with cell wall remodeling in the CV vs. CO comparison, we speculate that cells activate a cell wall compensatory pathway to strengthen the cell wall in order to mitigate the negative effects of cold stress, and AsA may protect cell wall integrity under cold stress. Our hypothesis is supported by the finding that OsVTC1-1 RNAi lines contain lower levels of AsA and cell-wall-related proteins, such as cellulose synthase, β-galactosidase, and expansin [53].

Trehalose is a non-reducing disaccharide sugar that is found in a large variety of organisms, including bacteria, yeasts, invertebrates, and plants [54]. Our transcriptome data analysis showed that GO terms related to trehalose biosynthetic and metabolic processes were enriched in the CV vs. CO comparison (Figure 4). Studies have shown that exogenous trehalose treatment improves cold stress tolerance in rapeseed and melon, and transgenic rice with enhanced trehalose levels confers high tolerance to different abiotic stresses [55,56,57]. In addition, transcriptome data showed that ABA-related genes, *NCED3* and *ABA2*, as well as genes involved in the phenylalanine metabolism pathway, such as *PAL1* and *PAL2*, have higher expression levels in the CV compared to CO (Table S3). The ABA and phenylalanine metabolism pathways have been shown to be involved in multiple stress responses [58]. Therefore, we infer that other DEGs that are differentially expressed in the CV vs. CO comparisons, such as genes related to trehalose, ABA, and PHE ammonia lyase, may also contribute to ascorbic-acid-mediated cold stress tolerance in tea plants.

## 4. Materials and Methods

### 4.1. Plant Materials and Cold Stress Treatments

The tea cultivar ‘Longjing 43’, which is grown in a field at the Tea Research Institute of the Chinese Academy of Agricultural Sciences, Hangzhou, China, was used in this study. Tea plants can be propagated by stem cutting, and tea branches are widely used as experimental material [59,60]. For the AsA and cold treatment experiments, the detached branches were initially kept in water for three days in a growth chamber under the following conditions: 6000 Lux photosynthetic photon flux density (PPFD), 14 h light and 10 h dark cycles, and 22 °C for daytime and 20 °C for night time. Then, the branches were sprayed with either Milli-Q water (Millipore-Sigma, Burlington, MA, USA), 1 mM AsA, or 5 mM AsA (A8100, Solarbio Life Science, Beijing, China) for two days. Twenty-four hours after the last AsA application, tea branches were kept at −3 °C for 2.5–3 h (cold stress) or in a 22 °C growth chamber (normal temperature control). After cold treatment, the young shoots with one bud and two leaves were taken for analysis of their relative electrolyte leakage, Fv/Fm, malondialdehyde (MDA), CAT enzyme activity, and transcriptome.

### 4.2. Electrolyte Leakage and Fv/Fm Measurement

Electrolyte leakage assays were performed, as previously described with modification [61]. Young shoots with one bud and two leaves were harvested before and after cold stress treatment, rinsed twice with Milli-Q water, placed into 50 mL tubes containing 20 mL of Milli-Q water, and shaken at 200 rpm for 2 h at room temperature before their electrical conductivity was measured (Orion 5 Star conductivity meter, Thermo Fisher Scientific, Waltham, MA, USA), giving S_1_. After detecting S_1_, the samples were boiled for 30 min and shaken at room temperature for another 1 h before their electrical conductivities were measured again, giving S_2_. The relative electrolyte leakage was calculated as: S_1_/S_2_ × 100 %.

For Fv/Fm measurement, branches were placed in the dark for 20 min. Then, the Fv/Fm was measured in the young shoots with one bud and two leaves by using a FluorCam 7 (Photon Systems Instruments, Banbury, UK). Five replicates were performed for each treatment.

### 4.3. MDA Content Measurement

To measure MDA content, 100 mg of the sample was homogenized in 1 mL of extraction solution and centrifuged at 8000× *g* for 10 min at 4 °C. The supernatant was collected and mixed with an MDA assay kit (MDA-1-Y, COMIN, Hangzhou, China). The mixture was kept at 95 °C for 30 min, cooled to room temperature, and then centrifuged at 10,000× *g* for 10 min. The absorbance of the supernatant at 532 nm and 600 nm was measured using a microplate reader (Molecular Devices, San Jose, CA, USA).

### 4.4. CAT Enzyme Activity Measurement

To measure CAT enzyme activity, 100 mg of materials were homogenized in 1 mL of acetone, centrifuged at 8000× *g* for 10 min at 4 °C, and the supernatant was collected. CAT activity was measured using a CAT activity assay kit (CAT-1-W, COMIN, China) according to the instructions provided by the manufacturer.

### 4.5. RNA Isolation, RNA-Sequencing, and Data Analysis

Total RNA was extracted using the RNAprep Pure Plant kit (DP441, TIAGEN, Beijing, China) and then treated with DNaseI (RT411, TIAGEN) to remove contaminating genomic DNA following the manufacturer’s instructions. The RNA integrity was assessed by the Bioanalyzer 2100 system (Agilent Technologies, Santa Clara, CA, USA). A total of 400 ng total RNA was used to prepare RNA-seq libraries using the NEB Next^®^ Ultra RNA Library Prep Kit for Illumina (E7530, NEB, Northborough, MA, USA). The library was sequenced on an Illumina Novaseq platform by the Novogene Company (Beijing, China), and 150 bp paired-end reads were generated for each sample.

Quality control of raw data was performed by Novogene Company (Beijing, China). Clean reads were obtained by removing the reads containing adapter, poly-N, and low-quality reads from the raw data and mapped to the ‘Longjing 43’ reference genome [62] by Hisat2 (v2.0.5) [63]. A novel transcripts prediction was performed using StringTie (v1.3.3b) [64]. FeatureCounts (v1.5.0) was used to count the reads numbers mapped to each gene [65], and then the FPKM of each gene was calculated based on the length of the gene and read count mapped to this gene. The DESeq2 R package (v1.20.0) was used to identify differentially expressed genes (DEGs) [66]. Those genes which had an FDR ≤ 0.05 were considered DEGs.

Tbtools was used for GO (Gene Ontology) functional analysis, and an online platform (https://www.bioinformatics.com.cn (accessed on 2 November 2022)) was utilized to graphically present the findings of the GO functional enrichment studies, with adjusted *p*-values of < 0.05 serving as the threshold.

### 4.6. Quantitative Real-Time PCR (qPCR)

A total of 1 μg of total RNA was used to synthesize first-strand cDNA using a PrimeScript RT reagent kit (RR047A, Takara, Dalian, China), following the manufacturer’s instructions. The qPCR assay was performed on the LightCycler 480 system with SYBR Green I Master Mix (4887352001, Roche Diagnostics GmbH, Mannheim, Germany). The *CsPTB* gene was used as the internal standard to normalize the cDNA concentrations [67]. The relative expression level of each gene was calculated using a 2^−ΔΔCt^ method [68]. Primers used for qPCR are listed in Table S1.

### 4.7. Statistical Analysis

All experiments were performed with at least three independent biological replicates. Data represent the mean ± SEM of biological replicates. Data were statistically analyzed using the Student’s *t*-test or two-way ANOVA analysis.

## 5. Conclusions

**Figure 8:**
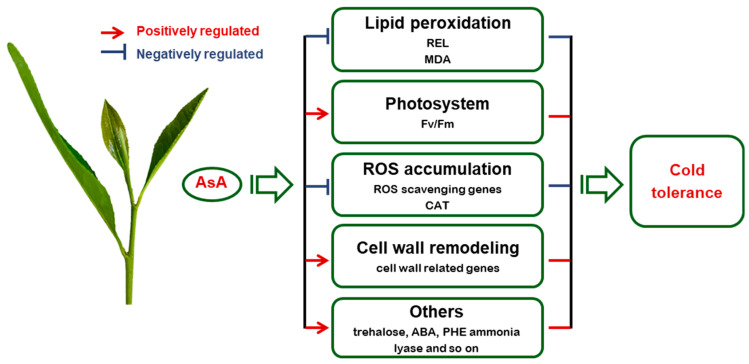
A proposed model of AsA enhancing cold stress tolerance in tea plants. AsA enhances the cold tolerance of the tea plant by decreasing the damage of lipid peroxidation, protecting the photosystem, and promoting the expression of cell-wall-related genes, and other genes related to stress response.

In summary, cold stress causes lipid peroxidation, reduces Fv/Fm, and increases the expression of ROS-scavenging genes in tea plants. We show that AsA has a protective role on tea plants against cold stress. AsA adversely regulates its biosynthesis and ROS scavenging, while simultaneously affecting the expression of cell-wall-related and other genes (Figure 8). These findings shed light on the role and regulatory mechanisms of AsA in the cold stress response of tea plants and may aid in the development of natural metabolic reagents for enhancing tea plants’ cold stress tolerance.

## Data Availability

All data generated or analyzed during this study are included in the published article and Supplementary Files.

## Supplementary Materials

The supporting information can be downloaded at: https://www.mdpi.com/article/10.3390/ijms241210059/s1.
